# Comparing Video-Based and Face-to-Face Psychotherapy: Systematic Review and Multilevel Meta-Analysis Across Mental Disorders

**DOI:** 10.2196/89177

**Published:** 2026-08-05

**Authors:** Christian Meyer-Keirath, Hannah Wallis, Mariebelle Kaus, Michael Schenk, Jolina Holzhaus, Caroline Rometsch, Claudia Buntrock, Christian Apfelbacher, Florian Junne

**Affiliations:** 1 University Clinic for Psychosomatic Medicine and Psychotherapy, University Medicine, Otto-von-Guericke-University Magdeburg, Medical Faculty, Leipziger Str. 44, Magdeburg, Saxony-Anhalt, 39120, Germany, 49 6714200; 2 Partnersite Halle-Jena-Magdeburg, Deutsches Zentrum für Psychische Gesundheit, Magdeburg, Sachsen-Anhalt, Germany; 3 Institute of Social Medicine and Health Systems Research, Medical Faculty, University Hospital Magdeburg, Magdeburg, Saxony-Anhalt, Germany; 4 Department of Psychosomatic Medicine and Psychotherapy, Universitätsklinikum Tübingen, Tübingen, Baden-Wurttemberg, Germany; 5 Center for Behavioral Brain Sciences, Otto-von-Guericke-University, Magdeburg, Saxony-Anhalt, Germany

**Keywords:** video-based psychotherapy, meta-analysis, internet-based treatment, telehealth, telemedicine

## Abstract

**Background:**

Video-based psychotherapy (VBT) is increasingly used to expand access to mental health care. Review studies generally report symptom outcomes similar to those achieved with face-to-face (F2F) psychotherapy. However, these studies often analyze VBT alongside other remote modalities, and it remains unclear what portion of the effects can be attributed to the video-based setting itself.

**Objective:**

The objective of this systematic review is to compare VBT and F2F psychotherapy in terms of symptom reduction, using strict methodological inclusion criteria, to maximize comparability between the delivery formats while also considering therapy duration.

**Methods:**

We searched bibliographic databases (MEDLINE via PubMed, PsycINFO, and Embase) from inception to May 20, 2025, supplemented by reference checking. The search was updated on March 10, 2026. We conducted a systematic review of randomized controlled trials (RCTs) comparing synchronous VBT with F2F psychotherapy in adults and reporting symptom severity. Trials had to meet a minimum treatment dose (≥500 min) and limited hybrid exposure (≤1/3 of sessions delivered F2F). Interventions had to be delivered by health professionals. We excluded interventions delivered solely via telephone or asynchronous platforms, blended formats, or unstructured counseling or group settings. Risk of bias was assessed following Metapsy (RoB2) guidelines, and certainty of evidence was rated using the Grading of Recommendations Assessment, Development and Evaluation (GRADE) approach. Standardized mean differences (Hedges *g*) of posttreatment symptom severity were synthesized using a 3-level correlated and hierarchical effects (CHE) random-effects model to account for dependent outcomes.

**Results:**

Out of 11,386 identified records, 87 articles underwent full-text review; 12 RCTs (465/900, 51.7% female; mean age 40.21, SD 14.52 y) met the inclusion criteria. Diagnoses included posttraumatic stress disorder, depression, obsessive-compulsive disorder, bulimia nervosa, generalized anxiety disorder, and somatoform pain. Across 41 effect sizes, no significant differences in symptom reduction emerged between VBT and F2F psychotherapy (Hedges g=–0.09, 95% CI –0.52 to 0.33; SE=0.19; *P*>.99). Between-study heterogeneity was substantial; the 95% prediction interval was −1.65 to 1.46. No moderating effects were detected. The Akaike information criterion favored the 3-level model over conventional approaches.

**Conclusions:**

The evidence base was limited in size and scope (predominantly Western settings, posttraumatic stress disorder diagnoses, and cognitive behavioral interventions). Study limitations, heterogeneity, and few noninferiority trials reduced the certainty of the evidence. This review is innovative in isolating synchronous VBT from other remote delivery formats to understand the specific impact of the setting. Unlike previous reviews, this one provides a more specific estimate of the effects of the delivery setting. This is achieved by isolating synchronous video delivery, applying dose/setting restrictions, and focusing on RCTs. Results refine the current evidence base and support offering VBT as a pragmatic delivery option to extend access to mental health care. Adequately powered noninferiority trials across diverse diagnoses, cultures, and longer-term treatments are needed.

## Introduction

### Background

The demand for psychotherapeutic treatment remains globally high, and care is still insufficient regarding treatment access, particularly in pandemic situations and emergencies [1]. The likelihood of receiving psychotherapy for patients experiencing severe mental disorders ranged from 49.7% to 64.5% in highly developed countries. During the COVID-19 pandemic, access to psychotherapeutic treatments was further restricted [2], while the need for psychotherapy was growing [3,4]. Access to psychological treatment is especially limited for vulnerable populations, such as residents of rural areas [5-7] and ethnic minorities [8]. In rural areas, the number of professionals offering mental health support is low, despite evidence of an increased need for mental health services [7].

In this review, we refer to synchronous video-based psychotherapy (VBT) as remote psychotherapy where patients and psychotherapists interact in real time using video conferencing technology similar to face-to-face (F2F) counseling and psychotherapy [9]. VBT overcomes geographic barriers and provides flexible service delivery [10]. The pandemic acted as a major catalyst for its widespread implementation [11], and VBT continues to be provided at levels above those observed prior to the pandemic [12], with high acceptance among digital mental health interventions [13]. Guidelines for VBT were established in 2013 by experts of the American Psychological Association [14] and the American Telemedicine Association [15].

Previous reviews suggest that VBT achieves outcomes comparable to F2F psychotherapy across several mental health conditions, with similar dropout rates and patient satisfaction [16-20]. Despite evidence of efficacy, substantial heterogeneity across study samples and critical methodological limitations restrict the strength of the evidence. In the field, it is debated that the wide variation in digital tools and methodologies used in video-based psychotherapy studies makes it difficult to generalize the findings [21]. Our study aims to contribute scientific evidence to this debate. Prior reviews have often addressed broader or different questions, for example, by synthesizing live psychotherapy by video across diverse clinical populations and treatment contexts [17], combining telephone- and video-based telehealth psychotherapy [18], focusing on specific disorders such as depression, anxiety-related conditions, or other selected clinical populations [22-26], examining therapeutic alliance rather than symptom outcomes [27], or mapping the broader field of synchronous web-based psychotherapy from a health quality perspective [28]. Prior evidence has not consistently differentiated between technical modalities (eg, telephone vs VBT) [18,19,29], and therapy duration is rarely controlled for, despite considerable variation across countries and health care systems (eg, up to 12 sessions in the United States vs 41 in Germany). As a result of these broader conceptualizations of VBT, it remains unclear to what extent observed effects reflect the specific impact of the video-based setting itself or other effects of digital psychotherapy. A more specific focus is needed to isolate the effect of the delivery format. In addition, therapy duration is rarely controlled for explicitly, which may further reduce comparability between delivery formats.

To address these gaps, we systematically reviewed and meta-analyzed randomized controlled trials (RCTs) comparing synchronous VBT with F2F psychotherapy in adults while excluding other digital modalities such as telephone-based, asynchronous, or guided self-help interventions. To improve comparability between delivery formats, we restricted inclusion to interventions with a minimum psychotherapy dose of 500 minutes, to exclude ultrabrief treatments. Because several studies reported multiple relevant outcomes, we applied a 3-level meta-analytic model to account for within-study dependence and to estimate heterogeneity across levels.

### Objectives

We hypothesized that VBT shows comparable efficacy to F2F psychotherapy in terms of symptom reduction. Specifically, we expected no significant differences in treatment outcomes between the 2 modalities.

## Methods

### Eligibility Criteria

The following inclusion and exclusion criteria were applied.

The inclusion criteria are as follows: (1) RCTs on adults (≥18 y) with a mental disorder diagnosed by established diagnostic criteria (ie, *International Classification of Diseases*, 10th Revision [*ICD-10*]; *Diagnostic and Statistical Manual of Mental Disorders, Fifth Edition* [*DSM-5*]); (2) a psychotherapy intervention of a total duration of at least 500 minutes divided into sessions of at least 25 minutes, to exclude ultra-brief interventions—psychotherapy had to be delivered in a synchronous VBT setting, and no more than one-third of the sessions was applied in a F2F setting; and (3) the intervention was delivered by health professionals (ie, psychological psychotherapists, psychologists, medical psychotherapists, doctors) or trainees (ie, in formalized psychotherapeutic training).

The exclusion criteria were as follows: (1) psychotherapy was applied as an adjunct to another treatment (eg, physical therapy, pharmacotherapy); (2) psychotherapy was delivered in the context of unstructured counseling sessions (<25 min at a time) or crisis interventions; (3) psychotherapy took place outside of psychotherapeutic settings (eg, occupational therapy, social counseling, etc); (4) psychotherapy was applied in a group setting; (5) missing reports of any of the following data: distribution of age, sex, diagnosis or indication for treatment, number and duration of treatment sessions, measures of dispersion for demographics, outcomes, and determinants/predictors.

### Information Sources

The databases PubMed, Embase, and PsycInfo (via ProQuest) were systematically searched from inception up to May 20, 2025. Each database was searched individually using its respective platform. Studies were restricted to English- and German-language publications; no additional restrictions were applied. Furthermore, reference lists of relevant reviews and included studies were screened manually to identify eligible studies. No study registries were searched, no additional online or print resources were browsed, and no authors or experts were contacted. The search was updated by rerunning the original database-specific search strategies on March 10, 2026, to identify newly published eligible studies. The reporting of the search strategy follows the PRISMA-S (Preferred Reporting Items for Systematic Reviews and Meta-Analyses Search extension; Checklist 1) [30] of the PRISMA 2020 guideline (Checklist 2) [31].

### Search Strategy

The search string combined vocabulary terms (eg, MeSH terms in PubMed) and free-text keywords related to psychotherapy, video-based delivery, and F2F comparators. Database-specific syntax was applied as appropriate. The complete database-specific search strings are provided in Multimedia Appendix 1. The search strategy was developed specifically for this review, without adaptation from a previously published review, and it was not formally peer-reviewed prior to execution.

### Selection Process

Identified records were imported into Rayyan.ai [32]. Duplicates were identified using the automated duplicated detection function of Rayyan and were subsequently checked manually. Four independent reviewers (CM-K, JH, Lara Annie Fangradt, and Lisa Gerdes) then systematically screened titles and abstracts regarding inclusion and exclusion criteria. Full texts of potentially eligible articles were then independently screened by the same 4 reviewers. Disagreements at any stage were resolved through discussion. The study selection process is summarized in the *Results* section following the PRISMA 2020 guidelines [30].

### Data Collection Process

Variables of interest included study characteristics (year, author, title, and country), patient characteristics (sex, mean age, and diagnosis), duration of treatment, treatment approach, and symptom severity outcomes (means and corresponding SDs). Data were extracted independently by 2 investigators (Lara Annie Fangradt and Lisa Gerdes), and disagreements were resolved by a third investigator (CM-K). In one study [33], these measures were not reported; however, effect sizes for posttreatment group comparisons were available and were therefore extracted instead.

### Outcomes

The primary outcome was symptom severity. When multiple assessments were associated with symptom severity (eg, applying assessment instruments such as the Clinician-Administered Post Traumatic Stress Disorder [PTSD] Scale for *DSM-5* or the PTSD Checklist for *DSM‐5* to assess PTSD symptoms), multiple outcomes were extracted.

### Risk of Bias Assessment

The risk of bias assessment was performed by 2 independent reviewers (CM-K and MK) using the Metapsy Risk of Bias Assessment Tool [34,35], an adapted version of the revised tool for assessing the risk of bias in randomized trials (RoB2) [36]. The assessment covered the following domains: randomization process, deviations from intended interventions, missing outcome data, measurement of the outcome, and selection of the reported result. The risk of bias was assessed separately for each outcome within each study. To determine the overall risk of bias for a study, the highest level of bias identified across all outcomes was used. The final rating for each study was categorized as low risk, some concerns, or high risk of bias. For visualization, a traffic-light plot was generated using the R (R Foundation for Statistical Computing) package *robvis* [37].

### Synthesis Methods

R version 4.3.3 with the *metafor* package was used for data analysis [38]. All models were fitted by restricted maximum likelihood estimation and compared by using the Akaike information criterion (AIC). Although not prespecified in the protocol, we applied a Bonferroni-Holm correction to maintain the correctness of inferences, given the number of moderator analyses conducted, and to maintain a global significance level of 5%. Accordingly, only adjusted *P* values will be displayed. An overview of the adjusted *P* values and their corresponding unadjusted *P* values is shown in Multimedia Appendix 2. Heterogeneity was investigated by using *I*^2^*, τ*^2^ statistics, and Cochran *Q* test. Additionally, we calculated 95% prediction intervals to complement heterogeneity statistics.

All studies included in the meta-analysis either reported data for multiple mental disorders or applied more than one assessment instrument for the same mental disorder. Outcomes were converted to Hedges *g* indices. All studies meeting the inclusion criteria provided sufficient data for effect size calculation; therefore, none were excluded from the quantitative synthesis due to missing values.

Positive effect sizes indicate greater efficacy for VBT compared to F2F therapy, whereas negative effect sizes favor the F2F setting.

To address the unit-of-analysis problem arising from nonindependent effect sizes [39], we used a correlated and hierarchical effects (CHE) model [40].

Given that the true within-study correlation between effect sizes is unknown, a constant correlation of *r*=0.40 was assumed with the application of a 3-level structure. The first level represents variation within each sample (eg, the aggregated effect sizes reported in the primary studies that represent the pooled individual responses to different questionnaires). The second level accounts for dependencies within studies due to the measurement of multiple outcomes, whereas the third level captures variation between studies, reflecting differences in research designs, populations, and contexts. Random effects were estimated at the study and outcome levels. The 3-level approach allowed moderator analyses at multiple levels, enabling exploration of variables contributing to both within-study and between-study heterogeneity. Statistical inference was conducted using *t*- and *F*-distributions, as recommended by Viechtbauer [38].

As part of the sensitivity analysis, a leave-one-out approach was applied to examine the individual influence of each study on the overall model and to identify potential outliers. For each iteration, Cook’s distance and changes in difference in betas (DFBETA) values were calculated. A Cook’s distance greater than 4/n (with n representing the number of studies) and DFBETA values below −1 or above +1 were considered indicative of influential studies.

To further investigate heterogeneity, an additional multilevel meta-regression was conducted with the sex distribution of patients, the duration of psychotherapy, mental disorder (dummy coded), and risk of bias (low, some concerns, and high) as moderators.

A funnel plot was generated, plotting the SE against the standardized mean difference, to visually assess potential small-study effects.

### Certainty Assessment

The certainty of evidence was assessed using the GRADE (Grading of Recommendations Assessment, Development and Evaluation) approach [41] and the GRADE Handbook [42]. Since all included studies were RCTs, the quality of evidence initially started at high and was subsequently evaluated across the following domains: risk of bias, publication bias, imprecision, inconsistency, and indirectness. GRADE assessments were independently conducted by 2 reviewers (CM-K and Lily Blum), and disagreements were resolved by discussion.

## Results

### Study Selection

**Figure 1.**
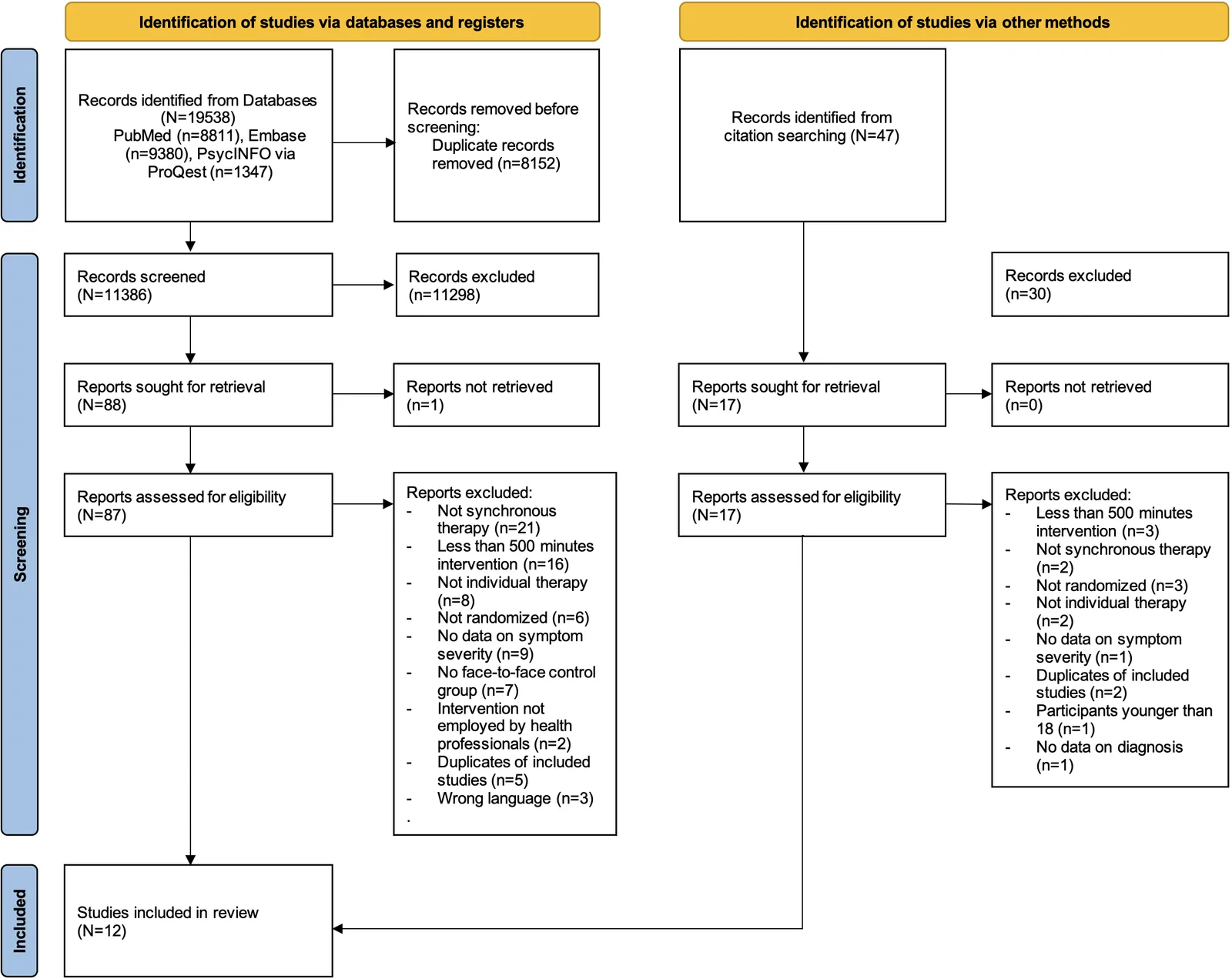
PRISMA (Preferred Reporting Items for Systematic Reviews and Meta-Analyses) flow diagram of the study selection process [33,43-53].

The database search and citation search yielded 19,538 records. After the removal of duplicates, 11,386 titles and abstracts were screened, and 87 articles underwent full-text review. Additionally, we assessed 17 articles from citation searching. The study selection process and reasons for exclusion at the full-text stage are presented in Figure 1.

### Study Characteristics

A total of 12 articles [33,43-53], published between 2008 and 2025, were included in the final analysis. Each study had independent participants, and no double-counting occurred. Across all articles, the total number of participants reached 900, with 51.7% (n=465) identifying as female. Participants’ mean age was 40.00 (SD 14.43) years in the experimental group and 40.42 (SD 14.61) years in the control group. Seven studies [33,43-48] were conducted in the United States, 2 in Iran [49,50], while 1 study each took place in Canada [53], Australia [51], and Japan [52]. Eleven studies [33,43-48,50-53] tested cognitive behavioral therapy (CBT), while one study examined intensive short-term dynamic psychotherapy [49]. Six studies [33,44-48] investigated patients diagnosed with PTSD. One study [43] each focused on patients with bulimia nervosa, medically unexplained pain [49], and generalized anxiety disorder [53]. An additional article assessed patients with depression, obsessive compulsive disorder, and generalized anxiety disorder [52]. One study did not report the specific diagnoses in the sample. The duration of the interventions varied significantly, ranging from 720 [33,47,51] to 1350 minutes [46] (mean 992.5 min, SD 208.42 min). An overview of study characteristics is provided in Multimedia Appendix 3.

### Risk of Bias Assessment

**Figure 2.**
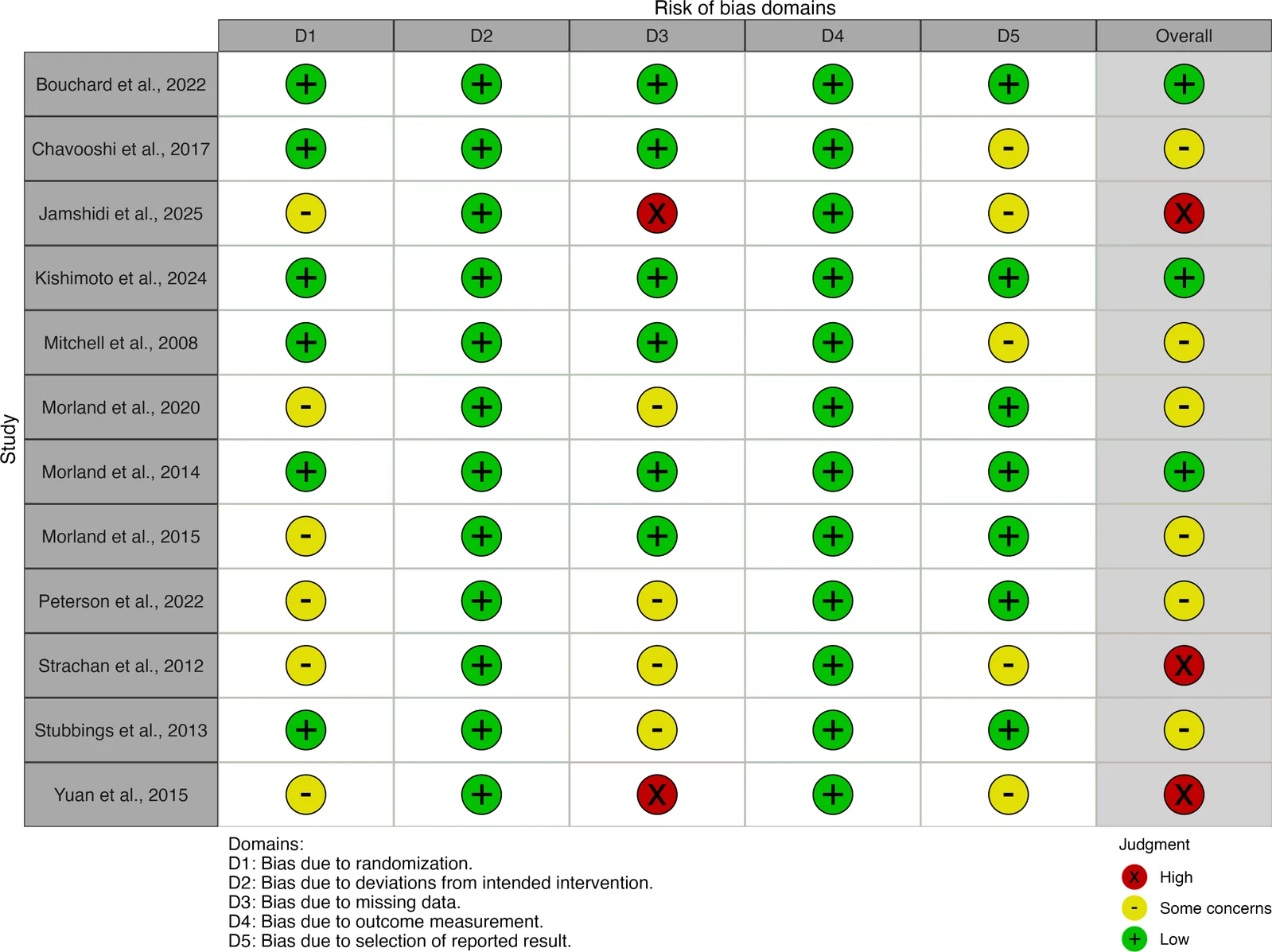
Risk-of-bias assessment of the included studies [33,43-53].

Of the included studies, 3 were rated as having a low risk of bias [44,52,53], 6 as raising some concerns [33,43,45,46,49,51]**,** and 3 as having a high risk of bias [47,48,50] (visualized in Figure 2). A moderator analysis (see Table 1) revealed no significant association between risk of bias and effect sizes.

**Table 1.** Intervention studies leave-one-out effect sizes and corresponding influence measures^a^.

Study	Cook distance	DFBETA^b^	Hedges *g*	*I*^2^ Level 1^c^	*I*^2^ Level 2^d^	*I*^2^ Level 3^e^
Bouchard et al [53], 2002	0.01	0.10	−0.12	12.48	21.61	65.91
Chavooshi et al [49], 2017	0.49	−1.82	0.03	43.89	45.23	10.89
Jamshidi et al [50], 2025	0.01	0.07	−0.11	11.55	16.12	72.33
Kishimoto et al [52], 2024	0.06	−0.25	−0.05	14.93	2.76	82.31
Mitchell et al [43], 2008	0.00	−0.01	−0.09	11.75	21.48	66.77
Morland et al [44], 2014	0.02	0.12	−0.12	12.38	16.96	70.65
Morland et al [45], 2015	0.01	0.10	−0.12	12.33	16.91	70.76
Morland et al [46], 2020	0.01	0.08	−0.11	12.13	17.78	70.09
Peterson et al [33], 2022	0.07	0.26	−0.15	12.56	18.09	69.35
Strachan et al [47], 2012	0.01	0.07	−0.11	11.50	17.55	70.94
Stubbings et al [51], 2013	0.05	0.21	−0.14	11.89	18.12	69.98
Yuen et al [48], 2015	0.00	0.05	−0.11	11.45	18.98	69.56

a The table shows the results of the leave-one-out sensitivity analyses, in which each study was removed sequentially from the model. Larger Cook’s distance and absolute difference in betas (DFBETA) values indicate greater influence of the respective study on the overall model estimate.

b DFBETA: difference in betas.

c *I*² Level 1 represents within-sample variance.

d *I*² Level 2 represents within-study variance across outcomes.

e *I*² Level 3 represents between-study variance.

### Results of Syntheses

**Figure 3.**
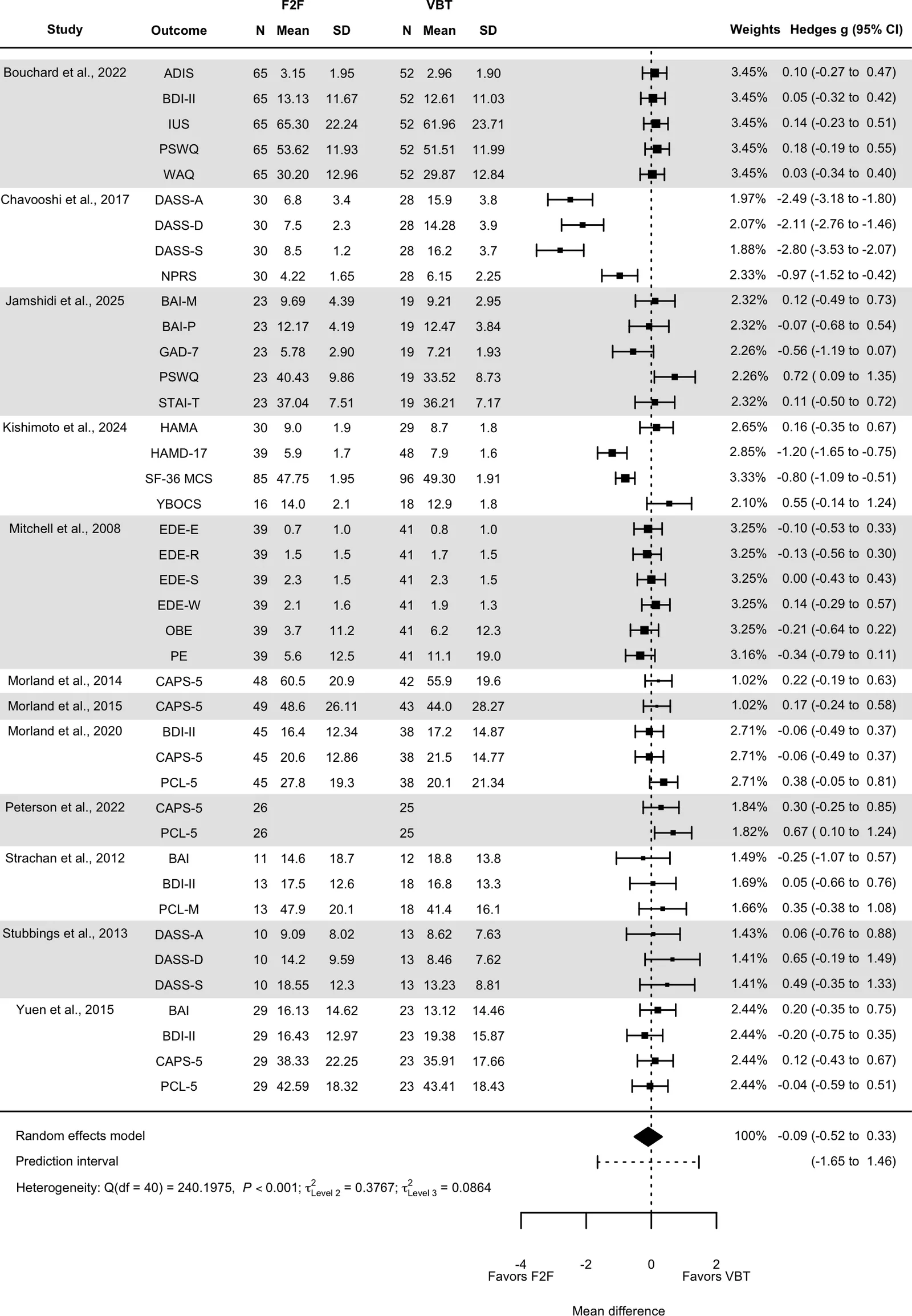
Forest-plot of effect sizes across all included studies [33,43-53]. ADIS: Anxiety Disorders Interview Schedule for DSM-IV; BAI: Beck Anxiety Inventory; BAI-M: Beck Anxiety Inventory-Mental; BAI-P: Beck Anxiety Inventory-Physical; BDI-II: Beck Depression Inventory-II; CAPS-5: Clinician-Administered PTSD Scale for *DSM-5*; DASS-A: DASS subscale anxiety; DASS-D: DASS subscale depression; DASS-S: DASS subscale stress; EDE-E: EDE subscale eating concerns; EDE-R: EDE subscale restraint; EDE-S: EDE subscale shape concerns; EDE-W: EDE subscale weight concerns; F2F: face-to-face psychotherapy; GAD-7: generalized anxiety disorder 7-item scale; HAMA: Hamilton anxiety rating scale; HAMD-17: Hamilton depression rating scale; IUS: intolerance of uncertainty scale; NPRS: numerical pain rating scale; OBE: objective binge-eating episode; PCL-5: PTSD checklist for *DSM-5*; PCL-M: PTSD checklist military; PE: purging episodes; PSWQ: Penn State Worry Questionnaire; SF-36 MCS: 36-item short-form health survey mental component summary; STAI-T: State-Trait Anxiety Inventory-Trait; VBT: video-based psychotherapy; WAQ: Worry and Anxiety Questionnaire; YBOCS: Yale-Brown obsessive compulsive scale.

A total of 41 outcomes were extracted, with an average of 3.42 extracted outcomes per study. The mean number of participants was 32.49 (SD 22.29) in the VBT condition and 33.22 (SD 21.59) in the F2F condition. The overall effect size of the 3-level model was Hedges *g*=−0.09 (95% CI -0.52 to 0.33; SE=0.19; *P*≥.99), indicating no statistically significant difference in posttreatment symptom reduction between the VBT and F2F settings (shown in Figure 3). The corresponding 95% prediction interval was −1.65 to 1.46, suggesting substantial variation in true effects across settings. The observed heterogeneity was statistically significant (*Q*_40_=240.20; *P*<.001).

In Figure 3, the estimates were derived from a 3-level CHE random effects model. Positive effect sizes indicate greater efficacy of the VBT setting. Squares represent individual study effects sizes weighted by inverse variance, with horizontal lines denoting 95% CIs. The diamond indicates the overall pooled effect estimate with its corresponding CI.

The study by Peterson et al [33] did not report means and corresponding SDs for posttreatment symptom severity but provided effect sizes for posttreatment group comparisons instead.

At the within-sample level (level 1), 11.97% of the total variance was explained, indicating that variations within individual samples contribute only marginally to the overall heterogeneity. At the within-study level (level 2), 16.42% of the total variance was explained, which likely reflects differences in outcome measures used within the articles with a heterogeneity of *I²*=16.42. Consequently, 65.60% of the total variance was explained at the between-study level (level 3) with a heterogeneity of *I²*=65.60. The corresponding estimated variance components were τ^2^_Level 2_=0.37 and τ^2^_Level 3_=0.09. The 3-level model exhibited an AIC value of 72.64, while the AIC value of the conventional model was 94.25. This suggests that our 3-level model outperforms the conventional model.

Overall, these results indicate that variance is least pronounced within individual samples (level 1), while differences within studies (level 2) are more substantial, likely due to the use of multiple outcome measures for the same construct. The vast majority of the variance was observed at the between-study level (level 3), likely reflecting differences in research designs, populations, or contexts. Consequently, further investigation of potential moderators at the between-study level is necessary.

### Analysis of Heterogeneity and Its Implications for the Interpretation of the Results

Detailed results of the influence diagnostics are reported in Table 1. The study by Chavooshi et al [49] exhibited both the highest Cook’s distance and the most extreme DFBETA values, indicating a substantial influence on the overall model estimates. However, the leave-one-out analysis confirmed the robustness of the model, as exclusion of any single study, including Chavooshi et al [49], did not meaningfully alter the overall estimate of Hedges *g*. Based on these findings, no study was excluded from the meta-analysis.

### Moderator Analysis

The meta-regression analyses did not identify any statistically significant moderators. Specifically, diagnostic category, therapeutic approach, sex, treatment duration, and risk of bias were not significantly associated with the comparative effects. An overview of the meta-regression results is presented in Table 2.

**Table 2.** Results of the moderator analysis and the corresponding statistics^a^.

Variable	n^b^	Hedges *g* (SE)^c^	*t* test (*df*)^d^	*P* value	95% CI
Duration	41	0 (0)	−2.09 (2.38)	>.99	0 to 0
Sex	41	0 (0)	−1.01 (1.41)	>.99	−0.02 to 0.02
PTSD^e^	21	0.73 (0.62)	1.18 (2.23)	>.99	−1.68 to 3.14
Anxiety	4	1.03 (0.49)	2.11 (1.21)	>.99	−0.81 to 2.87
MUP^f^	4	−1.63 (0.56)	−2.88 (1.72)	>.99	−3.67 to 0.41
Bulimia nervosa	6	0.62 (0.7)	0.89 (1.25)	>.99	−2 to 3.23
OCD^g^	1	1.56 (0.49)	3.18 (4.31)	>.99	−0.39 to 3.52
Depression	1	−0.19 (0.49)	−0.38 (4.31)	>.99	−2.14 to 1.77
GAD^h^	5	1.03 (0.76)	1.36 (4.61)	>.99	−1.97 to 4.02
Nonspecified diagnosis	4	0.39 (0.57)	0.69 (4.53)	>.99	−1.82 to 2.6
Risk of bias–some concerns	18	0.45 (0.41)	1.11 (2.08)	>.99	−1.84 to 2.74
Risk of bias–high	7	0 (0.42)	0.01 (2.74)	>.99	−2.09 to 2.1

a Positive estimates imply an advantage of VBT over in-person interventions.

b N:number of outcomes.

c *g: *Hedges *g*.

d df: degrees of freedom.

e PTSD: posttraumatic stress disorder.

f MUP: medically unexplained pain.

g OCD: obsessive-compulsive disorder.

h GAD: generalized anxiety disorder.

### Reporting Biases

**Figure 4.**
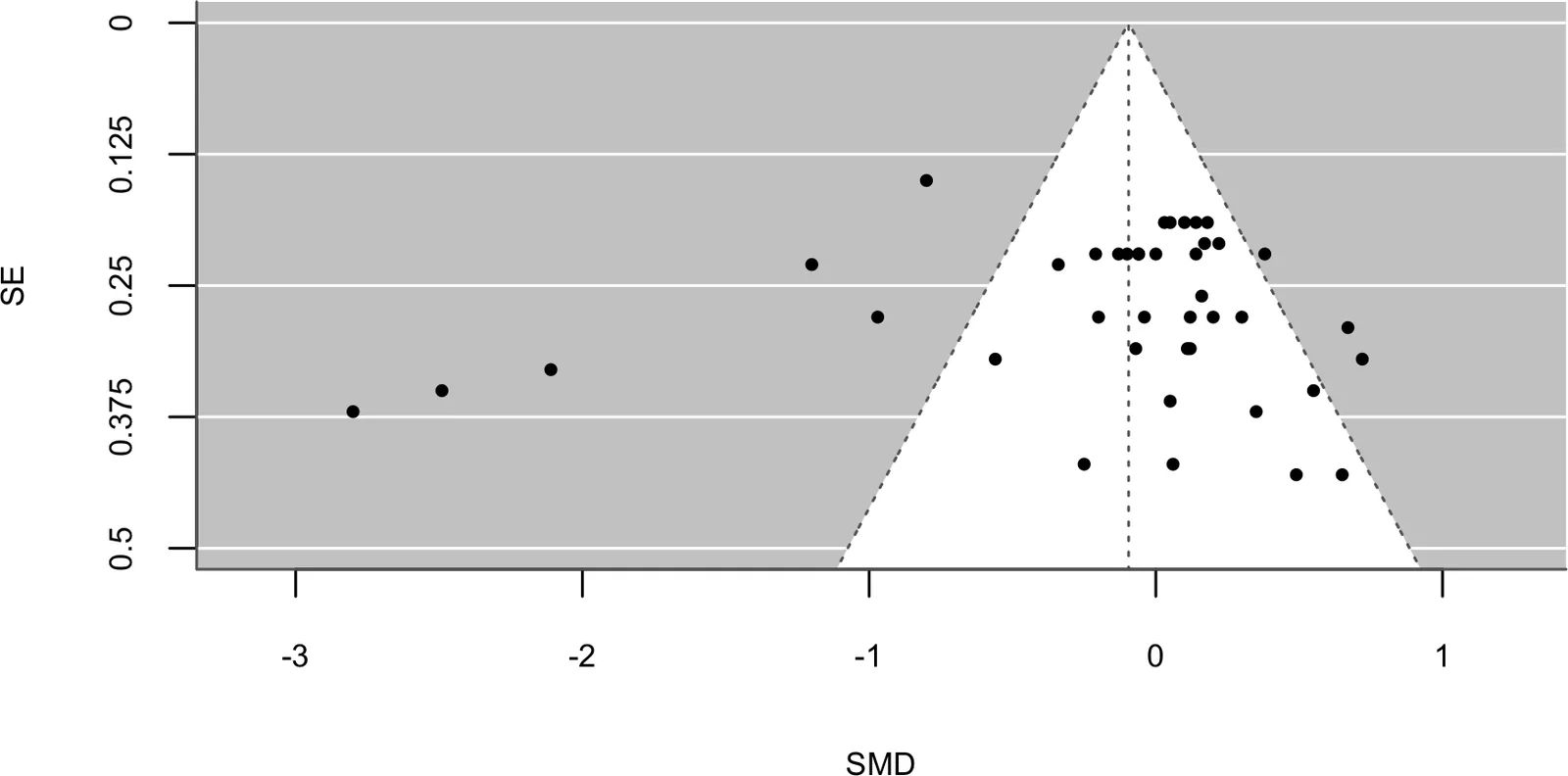
Funnel-plot of standardized mean differences for posttreatment symptom severity, displaying all individual outcome estimates from the included studies. SMD: standardized mean difference.

The funnel plot (see Figure 4) showed that articles were symmetrically clustered around the central effect size, particularly in regions where the standard error is higher (ie, smaller studies), indicating no small-study effects.

### Certainty of Evidence

According to the GRADE approach, the certainty of evidence for our primary outcome was rated as low. We applied one level of downgrading due to the results of our risk of bias assessment. Additionally, we downgraded another level for imprecision as the 95% CI of the overall effect size included both clinically important benefit and harm, based on a minimal important difference threshold of 0.2. No further downgrading was applied for the remaining domains. An overview of the certainty assessment can be found in Table 3.

**Table 3.** Grading of Recommendations Assessment, Development and Evaluation (GRADE) summary of findings^a^.

Outcomes	Anticipated absolute effects (95% CI)	Relative effect (95% CI)	Number of participants (studies)	Certainty of the evidence (GRADE)	Comments
Posttreatment symptom severity, SD units (Hedges g): investigators measured symptom severity using different instruments. Lower scores mean less symptoms	The posttreatment symptom severity in the F2F^b^ groups was on average 0.09 (SDs –0.52 to 0.33) lower than in the VBT^c^ groups	—	900 (11 RCTs)	⨁⨁◯ ◯ Low^d^^,e^	As a rule of thumb, 0.2 SD represents a small difference, 0.5 a moderate, and 0.8 a large

a GRADE Working Group grades of evidence. High certainty: we are very confident that the true effect lies close to that of the estimate of the effect. Moderate certainty: we are moderately confident in the effect estimate: the true effect is likely to be close to the estimate of the effect, but there is a possibility that it is substantially different. Low certainty: our confidence in the effect estimate is limited: the true effect may be substantially different from the estimate of the effect. Very low certainty: we have very little confidence in the effect estimate: the true effect is likely to be substantially different from the estimate of effect.

b F2F: face-to-face.

c VBT: video-based psychotherapy.

d Evidence limited by risk of bias.

e Evidence limited by imprecise data.

## Discussion

### Principal Findings

To our knowledge, this is the first meta-analysis that applied strict inclusion criteria designed to increase comparability between the delivery formats focusing on synchronous VBT vs F2F psychotherapy only. Consistent with our hypothesis, this systematic review and 3-level meta-analysis found no significant difference between VBT and F2F psychotherapy in posttreatment symptom severity. However, these findings should be interpreted in the context of substantial between-study heterogeneity, as demonstrated by our multilevel model, and the low certainty of evidence.

The results indicate that the minor and nonsignificant differences in posttreatment symptom severity are unlikely to be attributable to inherent differences between the 2 therapy settings.

This outcome broadly aligns with the findings of previous reviews on the subject [16,17,19] that support VBT as an alternative delivery format to traditional F2F psychotherapy. Extending previous reviews, we additionally calculated prediction intervals, which suggest that clinically relevant differences may still occur in specific contexts despite a nonsignificant average effect [54]. The substantial heterogeneity observed in previous reviews [16-18] further supports this interpretation. By applying a 3-level meta-analytic model, our study examined this heterogeneity in more detail and found that heterogeneity was predominantly located between studies rather than within studies. Together, these findings suggest that effects may vary across populations, treatment approaches, study designs, and health system contexts. This interpretation also echoes concerns raised by Steubl and Baumeister [21], who noted that the heterogeneity of digital tools and methodological approaches in VBT research limits the generalizability of previous findings.

The contribution of this meta-analysis is its methodological rigor and its strict inclusion criteria. Our strict approach allows for a more precise comparison of the two treatment modalities and ensures that the analysis mirrors the structure of traditional therapy as closely as possible, thereby providing a clearer understanding of the effects of the delivery format. To further enhance clinical relevance, we controlled for the effects of therapy duration. We applied a 500-minute minimum to ensure adequate therapeutic exposure, consistent with guideline recommendations [55,56] and dose-response evidence [57]. This threshold guaranteed that included interventions exceeded minimal adequacy, excluded ultra-brief treatments of limited comparability, and aligned with the lower bound of evidence-based treatment ranges. By including only RCTs, we attempted to ensure a high level of methodological rigor and to minimize the risk of bias, thereby enhancing the reliability of the findings. This rigorous selection process strengthens the overall validity of the conclusions drawn from the analysis.

### Limitations of Review Processes

Our rigorous approach has the drawback of confining the scope of the analysis to a limited number of studies and constraining the generalizability of the results. While there is a substantial body of literature on digital mental health interventions, much of it evaluates broader definitions of remote care, including telephone counseling, internet-based modules, or blended interventions rather than synchronous VBT alone [17,18,28]. In contrast, we deliberately restricted inclusion to trials delivering psychotherapy via synchronous video, consistent with established F2F practice. This improves treatment comparability but inevitably reduces the pool of available studies. Restricting the evidence base to RCTs also carries limitations. Although RCTs ensure high internal validity, they often involve highly selected samples and controlled conditions that may not reflect routine clinical practice. As a result, nonrandomized and naturalistic studies, which could provide important insights into real-world applicability and more diverse populations, were excluded. In addition, not all of the included trials were explicitly designed as noninferiority studies. As a result, the absence of significant differences between VBT and F2F psychotherapy cannot be equated with evidence of noninferiority.

### Limitations of Evidence

The included samples predominantly comprised Western populations, primarily from the United States, and were heavily weighted toward studies on PTSD, which is a well-researched topic with a substantial body of evidence and a relatively large sample size. However, most of the studies included in the analysis focused on highly specific populations, particularly US veterans and individuals exposed to war-related trauma. This restricts the applicability of the findings to other disorders, patient groups, and non-Western cultural contexts. Certain mental health conditions, such as somatoform, eating, or psychotic disorders, are also underrepresented in the literature, highlighting the need for broader exploration [17,18]. Furthermore, the therapeutic approaches studied were largely restricted to cognitive behavioral therapy, leaving an open question as to how well VBT integrates with other therapeutic modalities. There is also a lack of RCTs comparing VBT with F2F psychotherapy in long-term treatment [18].

One included study on chronic pain [49] found no pre-post effects for VBT, while the F2F setting demonstrated significant efficacy. However, this study differed from the others in several ways: It focused solely on chronic pain and was the only trial that conducted intensive short-term dynamic psychotherapy in a non-Western cultural setting. The authors attributed their findings to the absence of direct eye contact in VBT. However, it remains unclear whether the observed differences stem from the therapeutic approach, the diagnosis, or cultural factors. The outlier status of this study illustrates that heterogeneity across study designs and contexts may influence outcomes and limits generalizability of the average effects across different contexts.

Our assessment of small-study effects did not indicate clear evidence of publication bias, consistent with previous between-group analyses [16,17]. The moderator analysis showed that the risk of bias had no significant influence on the effect sizes, suggesting that methodological limitations had no systematic effect. However, given the small number of studies with low risk of bias, this finding should be interpreted with caution. This is further supported by the GRADE assessment, which rated the certainty of evidence as low. Similar concerns have been raised in previous reviews, which reported mixed study quality and methodological limitations in the available trials [18,19,58]. Future research should investigate whether more rigorous study designs yield comparable results.

### Future Research Directions and Practical Implications

This systematic review highlights the need for further research on the efficacy of VBT, employing standardized scientific criteria and direct comparisons with F2F psychotherapy. In particular, future studies should examine VBT and F2F psychotherapy in long-term therapeutic contexts. Additionally, it is imperative to expand research to encompass diverse cultural contexts, a broader range of mental health conditions, and a more diverse array of treatment approaches to fully realize the potential of VBT. Future trials should also be explicitly designed to test noninferiority, applying predefined noninferiority margins and adequate power calculations [59]. Such an approach would allow more robust conclusions about whether VBT can be considered truly noninferior to F2F psychotherapy.

From a practical perspective, the findings support synchronous VBT as a relevant delivery format for psychotherapy. At the same time, the question of the extent to which VBT can effectively address the shortage of mental health services, particularly in underserved or high-need regions, remains a critical one. Beyond structural and practical aspects, ethical considerations such as privacy, equitable access, and the preservation of therapeutic trust are equally crucial for sustainable implementation [60]. Further implementation research is therefore needed to clarify under which conditions VBT can be used most effectively and for which patients it is most appropriate.

### Conclusion

This review is innovative in providing a focused synthesis that isolates synchronous VBT from other remote delivery formats to understand the specific impact of the setting itself when the same psychotherapy is delivered via video instead of F2F. In contrast to broader previous reviews, this approach allows a more focused assessment of whether differences in outcomes are attributable to the video-based setting itself rather than to broader forms of telepsychotherapy. Under these more tightly controlled conditions, we found no clear evidence of a difference in posttreatment symptom severity between VBT and F2F psychotherapy. However, substantial between-study heterogeneity and low certainty of evidence highlight the need for adequately powered noninferiority trials and for further research on the contextual factors that may explain heterogeneity between studies. This contributes to the field by refining the current evidence base on telepsychotherapy and by highlighting the importance of treatment dose, study context, and methodological design when interpreting comparative outcomes. We draw from our findings that VBT can be regarded as a pragmatic delivery option to extend access to mental health care, while also leaving important questions open regarding its longer-term use and implementation. These include the implications of conducting psychotherapy exclusively via VBT over longer periods of time, as well as the need for targeted research and guidance on the specific challenges psychotherapists should consider when using VBT.

## Supplementary material

10.2196/89177 Multimedia Appendix 1 Database specific search strategies.

10.2196/89177 Multimedia Appendix 2 Original and adjusted *P* values for the included tests using Bonferroni-Holm correction for multiple comparisons.

10.2196/89177 Multimedia Appendix 3 Characteristics of included randomized controlled trials comparing synchronous video-based psychotherapy and face-to-face psychotherapy.

10.2196/89177 Checklist 1 PRISMA-S Checklist.

10.2196/89177 Checklist 2 PRISMA 2020 Checklist.

## Abbreviations

ADIS: Anxiety Disorders Interview Schedule
AIC: Akaike information criterion
CBT: cognitive behavioral therapy
CHE: correlated and hierarchical effects
DFBETA: difference in betas
F2F: face-to-face
GRADE: Grading of Recommendations Assessment, Development and Evaluation
PRISMA-S: Preferred Reporting Items for Systematic Reviews and Meta-Analyses Search
PTSD: posttraumatic stress disorder
RCT: randomized controlled trial
VBT: video-based psychotherapy
