# Supplementary material for: Comparing Video-Based and Face-to-Face Psychotherapy: Systematic Review and Multilevel Meta-Analysis Across Mental Disorders
Source: J Med Internet Res. 2026 Aug 5;28:e89177. doi: 10.2196/89177 (PMC13440640; doi:10.2196/89177)
Supplement: Supplementary file 3 [file jmir-v28-e89177-s001.docx]

**Supplementary Figure 1**

Database specific search strategies used in MEDLINE (PubMed), PsycINFO and Embase

| **PubMed** |  |
| --- | --- |
| Psychotherapy | ("Psychotherapy"[mesh] OR "Psychiatry"[mesh] OR "Psychotherapy"[tiab] OR "Therapy"[tiab] OR "Therapies"[tiab] OR "Psychotherap*"[tiab] OR "psychological treatment*"[tiab] OR "psychological intervention*"[tiab] OR "Psychiatr*"[tiab]) |
| Video-based delivery | ("Telemedicine"[mesh] OR "Telehealth"[tiab] OR "Telemedicine"[tiab] OR "Telepsychology"[tiab] OR "Video*"[tiab] OR "Online "[tiab] OR "internet therapy"[tiab] OR "web based therapy"[tiab] OR "Telepsychiatry"[tiab] OR "Telemental health"[tiab] OR "computer"[tiab] OR "Distance"[tiab] OR "Remote"[tiab] OR "videoconferenc*"[tiab] OR "videotherap*"[tiab] OR "telepsychotherap*"[tiab] OR "Telemedicin*"[tiab] OR "internet-based therap*"[tiab]) |
| Face-to-face delivery | ("face to face"[tiab] OR "face-to-face"[tiab] OR "in person"[tiab] OR "in-person"[tiab] OR "in-person-therapy"[tiab] OR "in-person-therapies"[tiab] OR "traditional"[tiab] OR "onsite"[tiab] OR "in-clinic"[tiab]) |

| **Embase** |  |
| --- | --- |
| Psychotherapy | ('Psychotherapy'/exp OR 'Psychiatry'/exp OR 'Psychotherapy':ti,ab OR 'Therapy':ti,ab OR 'Therapies':ti,ab OR 'Psychotherap*':ti,ab OR 'psychological treatment*':ti,ab OR 'psychological intervention*':ti,ab OR 'Psychiatr*':ti,ab ) |
| Video-based delivery | ('Telemedicine'/exp OR 'Telehealth':ti,ab OR 'Telemedicine':ti,ab OR 'Telepsychology':ti,ab OR 'Video*':ti,ab OR 'Online ':ti,ab OR 'internet therapy':ti,ab OR 'web based therapy':ti,ab OR 'Telepsychiatry':ti,ab OR 'Telemental health':ti,ab OR 'computer':ti,ab OR 'Distance':ti,ab OR 'Remote':ti,ab OR 'videoconferenc*':ti,ab OR 'videotherap*':ti,ab OR  'telepsychotherap*':ti,ab OR 'Telemedicin*':ti,ab OR 'internet-based therap*':ti,ab) |
| Face-to-face delivery | ('face to face':ti,ab OR 'face-to-face':ti,ab OR 'in person':ti,ab OR 'in-person':ti,ab OR 'in-person-therapy':ti,ab OR 'in-person-therapies':ti,ab OR 'traditional':ti,ab OR 'onsite':ti,ab OR 'in-clinic':ti,ab) |

| **PsycINFO (ProQuest)** |  |
| --- | --- |
| Psychotherapy | (MAINSUBJECT.EXACT.EXPLODE ("Psychotherapy") OR MAINSUBJECT.EXACT.EXPLODE ("Psychiatry") OR AB,TI("Psychotherapy") OR AB,TI("Therapy") OR AB,TI("Therapies") OR AB,TI("Psychotherap*") OR AB,TI("psychological treatment*") OR AB,TI("psychological intervention*") OR AB,TI("Psychiatr*") ) |
| Video-based delivery | (MAINSUBJECT.EXACT.EXPLODE ("Telemedicine") OR AB,TI("Telehealth") OR AB,TI("Telemedicine") OR AB,TI("Telepsychology") OR AB,TI("Video*") OR AB,TI("Online ") OR AB,TI("internet therapy") OR AB,TI("web based therapy") OR AB,TI("Telepsychiatry") OR AB,TI("Telemental health") OR AB,TI("computer") OR AB,TI("Distance") OR AB,TI("Remote") OR AB,TI("videoconferenc*") OR AB,TI("videotherap*") OR AB,TI("telepsychotherap*") OR AB,TI("Telemedicin*") OR AB,TI("internet-based therap*")) |
| Face-to-face delivery | (AB,TI("face to face") OR AB,TI("face-to-face") OR AB,TI("in person") OR AB,TI("in-person") OR AB,TI("in-person-therapy") OR AB,TI("in-person-therapies") OR AB,TI("traditional") OR AB,TI("onsite") OR AB,TI("in-clinic")) |
