# Supplementary material for: Comparing Video-Based and Face-to-Face Psychotherapy: Systematic Review and Multilevel Meta-Analysis Across Mental Disorders
Source: J Med Internet Res. 2026 Aug 5;28:e89177. doi: 10.2196/89177 (PMC13440640; doi:10.2196/89177)
Supplement: Supplementary file 4 [file jmir-v28-e89177-s002.docx]

| **Supplementary Table 2**  Original and adjusted p-values for the included tests using Bonferroni-Holm correction for multiple comparisons | | |
| --- | --- | --- |
| Test | Original p-value | Adjusted p-value |
| 3-level overall effect estimate | .629 | 1.000 |
| Cochran’s Q Test | <.001 | <.001 |
|  |  |  |
| Moderator analysis |  |  |
| Duration | .151 | 1.000 |
| Sex | .455 | 1.000 |
| PTSD | .348 | 1.000 |
| Anxiety | .151 | 1.000 |
| MUP | .080 | 1.000 |
| Bulimia nervosa | .456 | 1.000 |
| OCD | .077 | 1.000 |
| Depression | .738 | 1.000 |
| GAD | 0.298 | 1.000 |
| Non specified diagnosis | .556 | 1.000 |
| Risk of Bias – some concerns | .408 | 1.000 |
| Risk of Bias – high | .994 | 1.000 |
| *Note.*Original p-values and corresponding p-values after Bonferroni-Holm correction. PTSD = Posttraumatic Stress Disorder; MUP = Medically Unexplained Pain; OCD = Obsessive Compulsive Disorder; GAD = Generalized Anxiety Disorder | | |
