# Supplementary material for: Comparing Video-Based and Face-to-Face Psychotherapy: Systematic Review and Multilevel Meta-Analysis Across Mental Disorders
Source: J Med Internet Res. 2026 Aug 5;28:e89177. doi: 10.2196/89177 (PMC13440640; doi:10.2196/89177)
Supplement: Supplementary file 5 [file jmir-v28-e89177-s003.docx]

| **Supplementary Table 2**  Characteristics of Included Randomized Controlled Trials | | | | | | | | |
| --- | --- | --- | --- | --- | --- | --- | --- | --- |
| **Author (Year)** | **Country** | **Diagnoses** | **Sample Size¹** | **Mean Age (SD)** | **% Female (N)** | **Therapeutic Approach** | **Treatment Duration** | **Main Findings** |
| Bouchard et al. (2022) | Canada | GAD | VBT: 69/52 F2F: 79/65 | VBT: 41.35 (14.80) F2F: 39.38 (16.23) | 82.4 (122) | CBT | 900 Min | GAD symptoms decreased equally; videoconference CBT noninferior to F2F. |
| Chavooshi et al. (2016) | Iran | Somatoform Pain | VBT: 39/30 F2F: 42/28 | VBT: 31.87 (6.22) F2F: 32.2 (6.19) | 35.4 (26) | ISTDP | 960 Min | F2F ISTDP was significantly more effective than VBT in reducing pain and psychological symptoms in somatoform pain patients over 12 months |
| Jamshidi et al. (2025) | Iran | GAD | VBT: 23/19 F2F: 23/23 WLCC: 23/23 | VBT: 22.21 (1.75) F2F: 22.87 (1.89) WLCC: 21.70 (1.52) | 100 (69) | EMDR | 900 Min | Significant improvements in both treatment conditions compared to WLCC. No significant differences between treatment conditions, except for worry. Treatment effects remained stable at follow-ups. |
| Kishimoto et al. (2023) | Japan | Depression Anxiety OCD | VBT: 105/96 F2F: 94/85 | VBT: 39.7 (11.9) F2F: 40.7 (11.9) | 50 (91) | CBT | 1200 Min | No significant difference in outcomes; VBT noninferior to F2F for depression, anxiety, and OCD. |
| Mitchell et al. (2008) | USA | Bulimia Nervosa | VBT: 62/41 F2F: 66/39 | VBT: 28.4 (10.4) F2F: 29.6 (10.9) | 98.4 (126) | CBT | 1200 Min | CBT for bulimia via VBT was similarly effective and accepted as F2F. |
| Morland et al. (2014) | USA | PTSD | VBT: 57/42 F2F: 62/48 | VBT: 56.1 (11.8) F2F: 54.6 (13.2) | 0 (0) | CPT | 1080 Min | PTSD symptoms decreased equally in both groups. |
| Morland et al. (2015) | USA | PTSD | VBT: 63/43 F2F: 63/49 | VBT: 46.9 (11.8) F2F: 46.0 (12.1) | 100 (126) | CPT | 1080 Min | No significant difference in PTSD symptom reduction; both groups improved. |
| Morland et al. (2020) | USA | PTSD | VBT: 58/38 F2F: 58/45 | VBT: 47.3 (14.85)  F2F: 46.5 (12.82) | 26.7 (31) | PE | 1350 Min | All formats reduced PTSD symptoms equally; dropout lowest in F2F setting. |
| Peterson et al. (2022) | USA | PTSD | VBT: 44/25 F2F: 44/26 | VBT: 41.4 (8.6) F2F: 38.5 (11.8) | 11.3 (10) | CPT | 720 Min | PTSD symptoms improved in all formats; VBT showed larger effects than in-office F2F. |
| Strachan et al. (2012) | USA | PTSD | VBT: 22/18 F2F: 18/13 | 30.4 (7.7) | 7.5 (3) | BA-TE | 720 Min | PTSD and depression symptoms equally reduced in both settings. |
| Stubbings et al. (2013) | Australia | Non-specified | VBT: 14/13 F2F: 12/10 | 30 (11) | 57.7 (15) | CBT | 720 Min | No significant differences; CBT via video and in-person equally reduced anxiety, depression, and stress related symptoms. |
| Yuen et al. (2015) | USA | PTSD | VBT: 36/23 F2F: 38/29 | VBT: 41.2 (15.4 F2F: 46.3 (14.9) | 1.9 (1) | PE | 1080 Min | PE via VBT and F2F showed comparable symptom reductions and satisfaction. |
| ¹ Sample size indicates number of participants at pre-/post-treatment for each condition (Video-Based Therapy = VBT; Face-to-Face = F2F). Note: PTSD = Post traumatic stress disorder; OCD = Obsessive compulsive disorder; CBT = Cognitive behavioral therapy; ISTDP = Intensive short-term dynamic psychotherapy CPT = Cognitive Processing Therapy; PE = Prolonged Exposure; BT =  Behavioral treatment ; BA-TE = Behavioral activation and therapeutic exposure; WLCC = Waiting-List Control Condition | | | | | | | | |

| **Supplementary Table 2**  Characteristics of Included Randomized Controlled Trials | | | | | | |
| --- | --- | --- | --- | --- | --- | --- |
| **Author (Year)** | **Recruitment Period** | **Recruitment setting / location** | **Platform/Technology** | **Number of Sessions** | **Session frequency** | **Population** |
| Bouchard et al. (2022) | March 2016-December 2016 | University-based mental health clinics, Canada | Dedicated videoconferencing systems Standard: H.323 videoconferencing with Gatekeeper Hardware: Tandberg MXP90 videoconference codec systems, 32-inch TV monitor, external camera Secure IP connection (Bandwidth: 1.544 Mbps) | 15 sessions total | Weekly, each session 60 minutes  Same frequency and structure in both conditions | N = 148 adults Diagnosis: GAD 18-75 years Ethnicity: White Language: French-speaking |
| Chavooshi et al. (2016) | Not explicitly reported | Tehran, Iran Academic health centre clinics and community sources | Video teleconferencing via Skype Patients at home Devices: PC / Laptop / Tablet Internet: ≥2 Mbps | 16 sessions total | Weekly 60-minute sessions | N = 81 adults 18-45 years Condition: MUP Language: Farsi-speaking |
| Jamshidi et al. (2025) | Not explicitly reported | Persian Gulf University | Not explicitly reported “Web-based EMDR therapy was adapted for use in home-based telehealth” | 10 sessions total | Twice weekly | N = 65 females Undergraduate students from the Persian Gulf University |
| Kishimoto et al. (2023) | April 2021-February 2022 | 19 medical institutions providing psychiatric outpatient services in Japan | Live two-way video telepsychiatry Devices: Smartphones, Tablets, PC Patients at home  Patients chose platform 🡪 heterogeneous consumer platforms | Not fixed / not protocol-limited | Flexible | N = 199 adults Japan Standard outpatient psychiatric care (diagnoses: Depression, Anxiety, OCD and related disorders) |
| Mitchell et al. (2008) | October 1999-September 2003 | Rural and small urban communities in North Dakota and Minnesota, USA Recruitment methods: Mailings to local psychologists and physicians, media advertisements | Clinic-based telemedicine videoconferencing  System: Dedicated telemedicine units, T1 lines Patients attended sessions at local healthcare facilities, not from home 🡪 Clinic-to-clinic only  standardized camera and room layout | 20 sessions total | 20 sessions over 16 weeks, 1-2 sessions per week | N = 128 adults rural / semi-rural US population Diagnoses: Bulimia nervosa, EDNOS with binge eating/purging predominantly female, Caucasian |
| Morland et al. (2014) | March 2009-June 2013 | VA medical centers and Vet Centers across rural Hawaiian islands | clinic-based videoteleconferencing (Tandberg 880 VTC system) Patients at VA clinics (VA-standardized telehealth-network) | 12 sessions | Twice weekly, 90 min, 6 weeks total | N = 125 Male rural veterans with PTSD |
| Morland et al. (2015) | Not explicitly reported | VA clinical sites, Vet Centers, and community recruitment in Hawaii | Clinic-based videoteleconferencing  VA standardized videoconference systems | 12 sessions | Once or twice weekly, 90 min | N = 126 Civilian and veteran women with PTSD |
| Morland et al. (2020) | 2013-July 2018 | San Diego VA Medical Center and community-based outpatient clinics (California) | Home-based telehealth and clinc-based telehealth  VA-telehealth infrastructure, video-platforms VA-conform | Variable lengths (6-15 sessions) | Approximately weekly, 90 min | N = 175 Veterans with PTSD |
| Peterson et al. (2022) | September 2014-June 2018 | VA & DoD recruitment; veterans and active-duty personnel living within 60 miles of university clinics (USA) | Clinic-based /home-based CPT or home-based telehealth (videoconferencing) VA-telehealth system (HIPAA-conform) | 12 sessions | Weekly, 60 min | N = 120 Active-duty military and veterans with PTSD |
| Strachan et al. (2012) | December 2008-January 2010 | Large Southeastern VA medical center and affiliated university (USA) | Home-based telehealth videoconferencing via internet / landline early experimental home-based telehealth | 8 sessions | Weekly, 90 min | N = 40 OEF/OIF veterans with PTSD or subthreshold PTSD ± MDD |
| Stubbings et al. (2013) | January 2010-April 2011 | University psychology clinic, Perth, Western Australia | Clinic-based videoconferencing (Apple iChat) LAN-connection (Bandwidth: 100 Mbps)  controlled clinical environment | 12 sessions | Weekly, 60 mins | N = 26 Adults with mood and/or anxiety disorders |
| Yuen et al. (2015) | Not explicitly reported | Large Southeastern VA medical center & affiliated community-based outpatient clinics (USA) | Home-based telehealth videoconferencing  VA-telehealth equipment | 8-12 sessions | Weekly | N = 52 Veterans with combat-related PTSD |
